# Chemical Characterization and Antioxidant, Antimicrobial, and Anti-Inflammatory Activities of South Brazilian Organic Propolis

**DOI:** 10.1371/journal.pone.0165588

**Published:** 2016-11-01

**Authors:** Ana Paula Tiveron, Pedro Luiz Rosalen, Marcelo Franchin, Risia Cristina Coelho Lacerda, Bruno Bueno-Silva, Bruna Benso, Carina Denny, Masaharu Ikegaki, Severino Matias de Alencar

**Affiliations:** 1 Department of Agri-Food Industry, Food and Nutrition, “Luiz de Queiroz” College of Agriculture, University of São Paulo (USP), Avenida Pádua Dias, 11, 13418-900, Piracicaba, SP, Brazil; 2 Department of Physiological Sciences, Piracicaba Dental School, University of Campinas (UNICAMP), Av. Limeira, 901, CP 52, 13414-903, Piracicaba, SP, Brazil; 3 School of Pharmaceutical Sciences, Federal University of Alfenas, Rua Gabriel Monteiro da Silva, 714, Centro, 37130-000, Alfenas, MG, Brazil; Institute of medical research and medicinal plant studies, CAMEROON

## Abstract

South Brazilian organic propolis (OP), which has never been studied before, was assessed and its chemical composition, scavenging potential of reactive oxygen species, antimicrobial and anti-inflammatory activities are herein presented. Based on the chemical profile obtained using HPLC, OP was grouped into seven variants (OP1–OP7) and all of them exhibited high scavenging activity, mainly against superoxide and hypochlorous acid species. OP1, OP2, and OP3 had the smallest minimal inhibitory concentration (MIC) against Gram-positive bacteria *Streptococcus mutans*, *Streptococcus oralis*, and *Streptococcus aureus* (12.5–100 μg/mL). OP1, OP2, OP3, and OP4 were more effective against *Pseudomonas aeruginosa* (Gram-negative), with MIC values ranging from 100 to 200 μg/mL. OP6 showed anti-inflammatory activity by decreasing NF-kB activation and TNF-α release in RAW 264.7 macrophages, and expressing the NF-κB-luciferase reporter stable gene. Therefore, south Brazilian OP can be considered an excellent source of bioactive compounds with great potential of application in the pharmaceutical and food industry.

## Introduction

Numerous natural products have been widely used in folk medicine as therapeutic agents since the dawn of humankind. Almost all the ancient civilizations knew and used bee-derived products as valuable resources in their medicine. Among bee products, propolis stands out for having been traditionally used for centuries. Propolis, a food product, is a resinous substance collected by bees from plant buds and exudates in different regions of the world. Propolis was used by the Egyptians as one of the embalming components due to its antiputrefaction properties, by the Incas as an antipyretic agent, and from the seventeenth to the twentieth century it became very popular in Europe for its antibacterial activity. In the seventeenth century, it was already mentioned as an official drug in London pharmacopoeia [1,2]. Currently, a number of scientific studies have been proving its biological properties such as antioxidant, antinociceptive, anti-inflammatory, anticancer, antimicrobial, and anticariogenic activities [3–7].

Brazil is a great producer and exporter of propolis collected by *Apis mellifera*. In contrast to European propolis, rich in flavonoids, Brazilian propolis is characterized by the presence of derivatives of prenylated cinnamic acid [8,9]. Brazilian propolis has become popular as a healthy dietary supplement, and a number of innovative propolis-containing foods and beverages have been developed, because it has been proven to prevent inflammation, heart diseases, diabetes, and cancer [10,11]. Moreover, Brazilian certified organic propolis (OP), produced in environmental conservation and reforestation areas in the states of Paraná and Santa Catarina, has attracted the attention of Europeans due to its mild flavor and absence of heavy metals and pesticides. Nevertheless, not a single scientific study of the chemical profile and biological activities of certified OP has been carried out so far. Therefore, the aim of this study was to evaluate the chemical profile and antioxidant, antimicrobial, and anti-inflammatory activities of south Brazilian OP.

## Materials and Methods

### Chemicals

The following chemicals were used in this study: 2,2’-azino-bis(3-ethylbenzothiazoline-6-sulphonic acid) (ABTS), 2,2-diphenyl-1-picrylhydrazyl (DPPH), dihydrorhodamine 123 (DHR), sodium hypochlorite solution, β-nicotinamide adenine dinucleotide (NADH), phenazine methosulfate (PMS), nitroblue tetrazolium chloride (NBT), (+)-6-hydroxy-2,5,7,8-tetramethylchroman-2-carboxylic acid (Trolox^®^), 2,2’-azo-bis(2-amidinopropane) dihydrochloride (AAPH), resazurin, potassium persulfate, NaOCl, potassium phosphate buffer, phosphate buffer, and fluorescein disodium (Sigma-Aldrich, St. Louis, MO, USA); high-performance liquid chromatography (HPLC) analytical standards: acacetin, apigenin, 3,5-diprenyl-4-hydroxycinnamic acid (artepillin C), caffeic acid, chrysin, cinnamic acid, ellagic acid, ferulic acid, galangin, gallic acid, hesperetin, hesperidin, isorhamnetin, isosakuranetin, kaempferide, kaempferol, myricetin, *p*-coumaric acid, pinocembrin, quercetin, rhamnetin, rutin, sakuranetin, sinapic acid, tectochrysin, *tt*-farnesol, 2,4-dihydroxycinnamic acid, and vanillic acid (Extrasynthèse S.A., Genay, France); H_2_SO_4_ and all the solvents HPLC grade (J. T. Baker, Phillipsburg, NJ, USA); ethanol, Na_2_CO_3_, aluminum chloride, sucrose, and potassium acetate (Synth, Diadema, SP, Brazil); Folin-Ciocalteu reagent (Dinâmica Química Contemporânea, Diadema, SP, Brazil); brain-heart infusion (BHI) broth (BD-Difco, Becton, Dickinson and Company, Franklin Lakes, NJ, USA); defibrinated sheep blood (Vitrocell, Campinas, SP, Brazil); fetal bovine serum (FBS) (Gibco Life Technologies, Inc., Grand Island, NY), and ultrapure water, obtained from a Millipore Milli-Q System (Millipore SAS, Molsheim, France).

### Samples

A total of 78 samples of certified OP were collected in the following Brazilian municipalities: Bituruna (26°9'S, 51°33'W), Campo Largo (25°27'S, 49°31'W), Campo Magro (25°22'S, 49°27'W), General Carneiro (26°25'S, 51°19'W), Irati (25°28'S, 50°39'W), Mato Rico (24°42'S, 52°8'W), Pinhão (41°11'S, 7°32'W), Prudentópolis (25°12'S, 50°58'W), and União da Vitória (26°13'S, 51°5'W), in the state of Paraná; Canoinhas (26°10'S, 50°23'W), Papanduva (26°22'S, 50°8'W), Santa Terezinha (25°26'S, 54°24'W), Três Barras (26°7'S, 50°18'W), and Turvo (28°55'S, 49°41'W), in the state of Santa Catarina.

Sample collection was performed from February 2011 to January 2012 according to the rules of international certification of organic production and handling operations, namely National Organic Program (NOP) from the United States Department of Agriculture (USDA, processes no. 22422 and 23511), Kiwa BCS Öko-Garantie GmbH 2016 (processes no. A-2016-00005/2016-01341 and A-2016-00005/2016-01342) [12], and CEE (European) (processes no. 22422 and 23511) [12], as well as to Brazilian regulations (Orgânico Brasil, processes no. PR 106 and 12–0030) [13].

This study was carried out in private land after obtaining the owner permission. We guarantee that the field studies did not involve endangered or protected species.

### Preparation of extracts

OP samples were ground to a fine powder and 2-g aliquots (dry weight) were mixed with 25 mL of 80% (v/v) ethanol and shaken for 30 min at 70°C. After extraction, the mixtures were kept overnight at –18°C, centrifuged, and filtered using qualitative filter paper 14 μm pore size (J. Prolab, São José dos Pinhais, PR, Brazil) to produce the ethanolic extracts of propolis (EEP).

### Simultaneous determination of phenolic compounds and on-line HPLC-DAD-UV antioxidant activity

HPLC coupled with ABTS^•+^ assay was performed using the method developed by Koleva et al. [14] with some modifications. A stock solution containing 140 mM potassium persulfate and 7 mM ABTS^•+^ was prepared and kept at 25°C in the dark for 16 h to stabilize the radical. The radical reagent was prepared by diluting the stock solution in methanol to an absorbance of 0.700 ± 0.020 at 730 nm. The HPLC-separated analytes were first detected using diode-array detector (DAD) and, after post-column reaction coil with the preformed ABTS^•+^, the induced bleaching was detected as a negative peak by an ultraviolet (UV) detector at 734 nm. Results were expressed in antioxidant activity equivalent to Trolox (TEAC). HPLC separation was performed using reversed phase (RP)-HPLC in a chromatograph equipped with Shimadzu ODS-A column (RP-18, column size 4.6 × 250 mm; particle size 5 μm), DAD detector (SPD-M10AVp, Shimadzu Co., Kyoto, Japan), and ultraviolet-visible (UV-Vis) detector (SPD-20AV, Shimadzu).

The EEP were filtered through a 0.22 μm filter (Millipore) prior to injecting 20-μL aliquots into the HPLC system. The mobile phase consisted of water/acetic acid (99.5/0.5 v/v) (A) and methanol (100%) (B). Gradient elution was performed as follows: starting with 30% B and increasing to 40% B (15 min), 50% B (30 min), 60% B (45 min), 75% B (65 min), 75% B (85 min), 90% B (95 min), and decreasing to 30% B (105 min), at a solvent flow rate of 0.8 mL/min. HPLC eluates were detected using DAD detector prior to arriving at a T-junction, where ABTS^•+^ was added. ABTS^•+^ was delivered by a pump (LC-20AD vp, Shimadzu) at a flow rate of 0.8 mL/min. After the eluates were mixed with ABTS^•+^ in a reaction coil (15 m × 0.25 mm i.d. PEEK tubing), the negative peaks were measured using a UV-Vis detector at 734 nm. Data were analyzed using Shimadzu software Class-VP.

The detection limits (LOD) of artepillin C, gallic acid, caffeic acid, *p*-coumaric acid, and pinocembrin were 0.558, 0.041, 0.025, 0.0135, and 0.001 μg/mL, respectively, whereas their quantification limits (LOQ) were 1.69, 0.124, 0.074, 0.041, and 0.03 μg/mL, respectively.

### Total polyphenol and flavonoid contents

Total polyphenol contents (TPC) in EEP were determined using the Folin-Ciocalteu method [15] adapted to a microplate reader. The EEP (20 μL) were mixed with 100 μL of Folin-Ciocalteu reagent (1:10) and 75 μL of 7.5% Na_2_CO_3_. Absorbance was measured using a microplate reader SpectraMax^®^ M3 (Molecular Devices, LLC, Sunnyvale, CA, USA) at 740 nm after 40 min incubation at room temperature in the dark. A calibration curve was plotted using gallic acid as standard and the results were expressed as mg of gallic acid equivalents per gram of sample (mg GAE/g).

Total flavonoid contents in EEP were determined using the method described by Alencar et al. [16] with minor modifications. Aliquots of 0.5 mL of EEP were mixed with 4.3 mL of 80% ethanol, 0.1 mL of 10% aluminum chloride, and 0.1 mL of 1 M potassium acetate. After 40 min at room temperature, the absorbance was measured at 415 nm. A calibration curve was constructed using quercetin as standard and the results were expressed as mg of quercetin equivalents per gram of sample (mg QE/g).

### Scavenging of synthetic free radicals and reactive oxygen species (ROS)

#### DPPH free radical scavenging assay

Determination of DPPH free radical scavenging activity followed the method described by Moraes-de-Souza et al. [17] with some modifications. Aliquots of 66 μL of the standard, control, or EEP and 134 μL of 150 μM ethanol solution of DPPH were transferred to microplate wells. After 45 min in the dark, the absorbance was measured at 517 nm in a microplate reader SpectraMax^®^ M3. Ethanol was used as blank and a calibration curve was constructed with Trolox as standard, at concentrations ranging from 20 to 140 μM. The results were expressed as μmol Trolox equivalents per mg of sample (μmol TE/mg).

#### ABTS free radical scavenging assay

The antioxidant capacity was determined by free radical ABTS according to Al-Duais et al. [18] with modifications. The ABTS stock solution radical was diluted with 75 mM potassium phosphate buffer (pH 7.4). Aliquots of 20 μL of Trolox or EEP and 220 μL of ABTS radical solution were transferred to microplate wells and kept at room temperature in the dark. The absorbance was measured exactly 6 min after starting the oxidation. Potassium phosphate buffer was used as blank and Trolox was employed as standard at concentrations ranging from 12.5 to 200 μM. The results were expressed as μmol Trolox equivalents per mg of sample (μmol TE/mg).

#### Peroxyl radical scavenging assay

Peroxyl radical (ROO•) scavenging capacity was determined according to Melo et al. [19] with alterations. This assay was employed to monitor the antioxidant action of the EEP on the fluorescence decay by ROO•-induced oxidation of fluorescein and expressed as the oxygen radical absorbance capacity (ORAC). Aliquots of 30 μL of the standard, control, or EEP, 60 μL of 508.25 nM fluorescein disodium, and 110 μL of a solution of 76 mM AAPH were transferred to a microplate. The solutions were diluted with 75 mM potassium phosphate buffer (pH 7.4), also used as blank. The reaction was performed at 37°C and the absorbance of the samples was measured every minute for 2 h at 485 and 528 nm, the wavelengths of excitation and emission, respectively, using a microplate reader SpectraMax^®^ M3. Trolox was used as standard at concentrations ranging from 12.5 to 400 μM and the results were expressed as μmol Trolox equivalents per mg of sample (μmol TE/mg).

#### Superoxide radical scavenging assay

The capacity of OP to scavenge superoxide radical (O_2_•^–-^), generated by the NADH/PMS system, was determined according to Melo et al. [19] with modifications. For a final volume of 300 μL, 498 μM NADH, 129 μM NBT, different concentrations of EEP, and 16.2 μM PMS were added and dissolved in 19 mM potassium phosphate buffer (pH 7.4). The assay was conducted at 25°C and after 5 min the absorbance was measured at 560 nm. A control was prepared replacing the EEP with buffer and a blank was prepared for each sample dilution replacing PMS and NADH with buffer. The results were expressed as IC_50_, i.e. the mean quantity (μg/mL) of EEP required to quench 50% of the superoxide radicals.

#### Hypochlorous acid scavenging assay

Hypochlorous acid (HOCl)-scavenging capacity was measured by monitoring the effects of EEP on HOCl-induced oxidation of DHR to rhodamine 123, according to Gomes et al. [20] and Melo et al. [19] with modifications. HOCl was prepared using a 1% NaOCl solution, adjusting the pH to 6.2 by adding 10% H_2_SO_4_ solution. The reaction mixture contained EEP at different concentrations, 100 mM phosphate buffer (pH 7.4), 1.25 μM DHR, and 5 μM HOCl, in a final volume of 300 μL. The assay was conducted at 37°C in a microplate reader SpectraMax^®^ M3 and the fluorescence was measured immediately using the emission wavelength of 528 ± 20 nm with excitation at 485 ± 20 nm. The results were expressed as IC_50_ (μg/mL) of EEP.

### Antimicrobial activity

#### Minimal inhibitory concentration (MIC) and minimal bactericidal concentration (MBC)

The microorganisms used in this study were *Streptococcus mutans* UA159, *Streptococcus oralis* ATCC10557, *Streptococcus sobrinus* 6715, *Staphylococcus aureus* ATCC 25923, *Pseudomonas aeruginosa* ATCC 25619, and *Escherichia coli* ATCC 25922. The methodology described in BCSA [21] was modified into a microtechnique, in which 190 μL of BHI broth with inoculum (1–2 × 10^5^ cfu/mL) and 10 μL of EEP or control solution were dispensed onto a microplaque.

To determine MIC, the concentrations of EEP ranged from 12.5 to 1,600 μg/mL, and bacterial growth was assessed by adding 0.01% resazurin stain. Ethanol (4%) was used as the control. MIC values were defined as the lowest concentration of a given EEP that could inhibit bacterial growth.

To determine MBC, 30-μL aliquots of each incubated tube at concentrations higher than the MIC were cultured on BHI agar supplemented with 5% defibrinated sheep blood for 18–24 h, at 37°C, under atmospheric conditions of 5% CO_2_. MBC was determined as the lowest concentration that allowed no visible bacterial growth in agar [22].

#### Biofilm formation

In order to evaluate the antimicrobial activity of OP against the formation of *S*. *mutans* biofilm, the EEP were placed, at different concentrations (25–800 μg/mL), in the wells of sterile polystyrene U-bottom microtiter plates, previously treated with saliva. *S*. *mutans* cells (1 × 10^7^ cells/mL in BHI medium) were added to the wells containing BHI medium with 1% sucrose and these samples were incubated at 37°C for 18 h and 5% CO_2_. Biofilm growth was revealed and quantified using the crystal violet staining method and measuring absorbance at 575 nm [23,24]. After 18 h incubation, the spent medium was aspirated, non-adhered cells were removed, the wells were washed three times with sterile distilled water, and the plates were dried for 45 min before carrying out biofilm quantification [24].

### Anti-inflammatory activity

#### MTT assay

Cell viability was assessed using a modified 3-(4,5-dimethylthiazol-2yl)-2,5-diphenyl-2H-tetrazolium bromide (MTT) assay in RAW 264.7 macrophages (2 × 10^5^ cells/well). After 24 h of plating, the cells were treated with OP1–OP7 (0.1, 1, 10, and 100 μg/mL) for an additional period of 24 h. The absorbance was measured at 540 nm with a microplate reader (ASYS, UVM340, Biochrom, UK). The results were expressed as percentage of viable cells. The control group was considered as 100% cell growth.

#### NF-kB activation

RAW 264.7 macrophages were cultured and plated in 24-well plates, treated with EEP (0.1, 1, and 10 μg/mL) for 30 min and stimulated with lipopolysaccharide (LPS) (100 ng/mL) for 4 h. After that, luciferase assay reagent was added and luminescence emission was measured using a microplate reader (FlexStation 3, Molecular Devices, Sunnyvale, CA, USA) [25,26]. The results were expressed as mean ± SD of relative luminescence units (%). The control group was considered as 100% luminescence.

#### TNF-α quantification

RAW 264.7 macrophages were cultured and plated in 96-well plates under the same conditions used in the NF-kB activation assay. TNF-α was determined using ELISA according to the manufacturer’s instructions (R&D Systems, Minneapolis, MN, USA). The results were expressed in pg/mL.

### Statistical analyses

All determinations were carried out in triplicate and the results were expressed as means ± SD. Statistical analyses were performed using the Tukey’s test. The results were considered statistically significant when *p* < 0.05. Pearson’s correlation test was employed to determine the correlation between antioxidant capacity against synthetic free radicals and ROS.

## Results and Discussion

### Chemical characterization of south Brazilian OP

**Fig 1:**
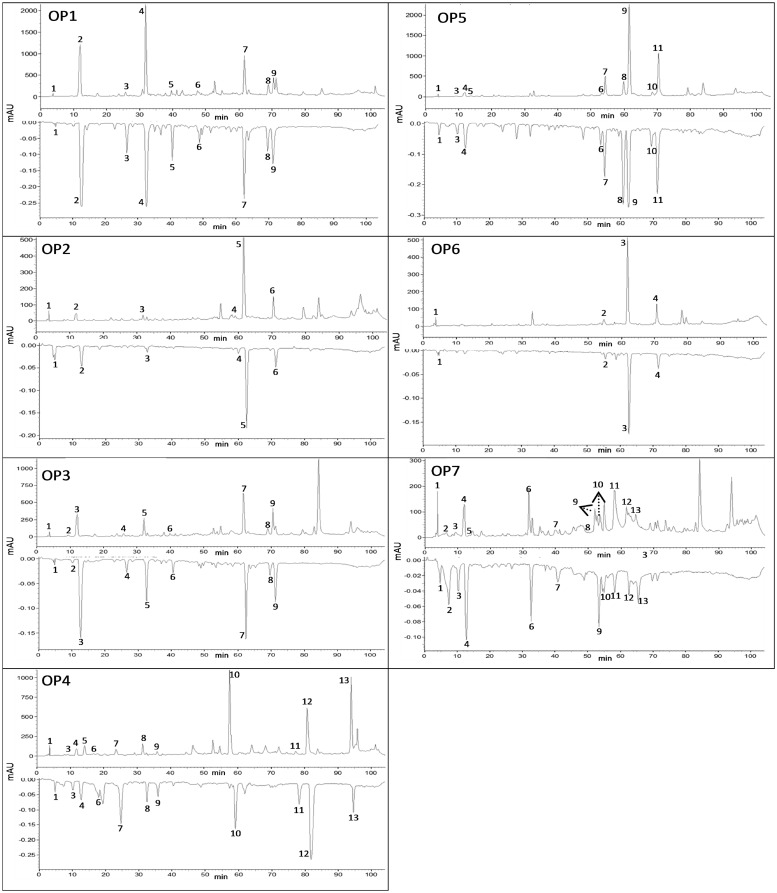
HPLC chromatograms of seven variants of organic propolis (OP1–OP7) detected at 260 nm (positive peaks) and 734 nm (negative peaks). Peak numbers refer to Table 1.

All OP samples used in this research have an international certification. This is the first time that propolis produced under organic conditions has been characterized. First of all, the 78 OP samples were analyzed using the high performance thin layer chromatography (HPTLC) and HPLC techniques [27,28], and based on these results they were grouped into seven variants (OP1–OP7), according to the qualitative chemical profile and on-line antioxidant activity using ABTS free radical scavenging (Fig 1).

Among the available standard compounds commonly found in propolis, from Brazil or from regions of temperate climate, in all the seven variants of OP we identified only three phenolic acids (gallic acid, caffeic acid, and coumaric acid), one prenylated derivative of cinnamic acid (artepillin C), and one flavonoid (pinocembrin). A common characteristic of all OP variants was the presence of gallic acid at concentrations ranging from 317.8 to 2,115.9 μg/mL. Despite the high concentration of gallic acid in OP7, the antioxidant activity of this compound reached only 102.6 μM TEAC (Table 1). Although coumaric acid was identified in OP4, OP5, and OP7, it was not responsible for the antioxidant activity and the latter did not have a detectable antioxidant activity.

**Table 1:** Characterization of individual peaks with antioxidant activity of seven variants of Brazilian organic propolis (OP1–OP7).

OP1			OP2			OP3			OP4			OP5			OP6			OP7		
Peak No.^a^	Content (μg/mL)	TEAC^b^ (μM)	Peak No.	Content (μg/mL)	TEAC (μM)	Peak No.	Content (μg/mL)	TEAC (μM)	Peak No.	Content (μg/mL)	TEAC (μM)	Peak No.	Content (μg/mL)	TEAC (μM)	Peak No.	Content (μg/mL)	TEAC (μM)	Peak No.	Content(μg/mL)	TEAC (μM)
1 (gallic acid)	482.7±0.5E	14.7±2.7	1 (gallic acid)	390.3±1.2F	85.6±15.7	1 (gallic acid)	643.5±3.1C	13.2±2.2	1 (gallic acid)	865.7±0.2B	80.3±3.9	1 (gallic acid)	547.5±0.1D	82.3±9.4	1 (gallic acid)	317.8±0.02G	26.2±0.6	1 (gallic acid)	2115.9±10.1A	102.6±32.1
2	–^c^	784±50.7	2	–	141.4±6.8	2	–	26.9±5.4	3 (caffeic acid)	355.3±0.5C	85.7±12	3 (caffeic acid)	466.5±0.2B	97.2±7.3	2	–	34.6±3.3	2	–	358.6±70.6
3	–	179.2±5.6	3	–	18.2±2.3	3	–	526.7±58.4	4	–	206.0±7	4	–	295.7±8.0	3	–	515.7±56.6	3 (caffeic acid)	716.1±2.9A	85.4±17.5
4	–	668.1±55.6	4	–	43.6±14.6	4	–	69.4±2.2	5 (coumaric acid)	11635.0±12A	–	5 (coumaric acid)	1621.0±3.7B	–	4	–	73.8±7.2	4	–	370±65.02
5	–	192.1±7.7	5	–	445.1±3.9	5	–	235.2±48.6	6	–	181.7±77.3	6	–	136.7±37.6				5 (coumaric acid)	883±8.2	–
6	–	131.3±36.6	6	–	82.7±1.1	6	–	69.0±2.3	7	–	415.0±33.7	7	–	331.2±8.0				6	–	179.2±28.8
7	–	529.1±12.4				7	–	451.7±59.8	8	–	127.0±3.8	8	–	550.4±59.2				7	–	115.1±42.3
8	–	156.3±37.2				8	–	76.8±12.8	9	–	100.5±7.8	9	–	726.4±91.3				8 (pinocembrin)	669.9±2.7	–
9	–	293.8±59.1				9	–	260.8±64.0	10	–	355.6±17.0	10	–	149.1±4.8				9	–	204.6±56.9
									11	–	216.7±11.0	11	–	471.3±9.6				10	–	86.2±17.3
									12 (artepillin C)	22.3±5.1	770.0±7.9							11	–	76.3±14.9
									13	–	174.8±7.6							12	–	143.7±35.4
																		13	–	66.5±10.2

^a^ Peak numbers are independent for each OP variant and refer to Fig 1.

^b^ TEAC: antioxidant activity equivalent to Trolox.

^c^ –: not identified.

Values represent the means ± standard deviation of triplicates; means followed by different uppercase letters in the same column differ statistically (*p* < 0.05) using the Tukey’s test.

OP4 presented high content of artepillin C (22,303 μg/mL of EEP), and this compound exhibited the highest antioxidant activity in this sample (Table 1). Artepillin C, found in the plant *Baccharis dracunculifolia*, the main source of green propolis [29], is known for its high antioxidant activity. Thus, the contribution of this plant species as the source of OP4 is noticeable. The variants of OP herein studied were produced in native areas and in environmental conservation and reforestation areas, which house a huge diversity of resin-producing plant species. Hence, it is reasonable that the chemical compounds found in these variants of OP not only vary abundantly, but also present differences in phenolic compounds commonly found in other propolis from Brazil as well as from other parts of the world. Additionally, many other ABTS free radical scavenging active compounds were detected in all variants of OP tested (Table 1).

Total flavonoid and phenolic contents are shown in Table 2. Phenolic compound contents ranged from 17.59 mg GAE/g (OP6) to 79.84 mg GAE/g (OP1), and the results obtained for all variants of OP were statistically different. In Brazil, green propolis produced in the state of Minas Gerais had 120 mg GAE/g [8], whereas red propolis collected in the state of Alagoas had 232 mg GAE/g [16]. These differences are mainly due to the types of plant species and ecosystem available for producing propolis.

**Table 2:** Total phenolic content, flavonoid content, and scavenging activity of seven variants of Brazilian organic propolis (OP1–OP7).

OP	Total phenolic	Flavonoid	ABTS^●+^	DPPH^●^	ROO^●^	O_2_^●-^	HOCl
	(mg GAE/g)	(mg QE/g)	(μmol TE/mg)			IC_50_^a^ (μg/mL)	
**1**	79.84±1.61^A^	0.33±0.001^B^	1.02±0.02^B^	0.30±0.01^B^	1.95±0.10^A^	1.05±0.05^B^	0.11±0.005^B^
**2**	67.02±0.66^C^	3.10±0.19^A^	1.05±0.03^B^	0.32±0.01^B^	1.48±0.11^B^	1.57±0.3^B^	0.04±0.01^B^
**3**	52.33±1.92^E^	nd^b^	0.81±0.05^C^	0.26±0.01^C^	1.17±0.12^C^	1.44±0.01^B^	0.06±0.005^B^
**4**	74.26±1.73^B^	nd	1.24±0.02^A^	0.38±0.01^A^	1.22±0.02^C^	0.61±0.01^C^	0.03±0,005^B^
**5**	59.78±1.60^D^	nd	0.80±0.003^C^	0.31±0.02^B^	1.58±0.10^B^	0.29±0.11^C^	0.05±0.01^B^
**6**	17.59±0.77^G^	nd	0.29±0.01^E^	0.01±0.0004^E^	0.50±0.07^D^	2.91±0,04^A^	1.45±0.15^A^
**7**	29.37±0.86^F^	3.03±0.09^A^	0.51±0.004^D^	0.10±0.002^D^	0.52±0.06^D^	1.75±0.04^B^	0.07±0.005^B^

^a^ IC_50_: inhibitory concentration to decrease by 50% the amount of reactive species in the tested media (mean ± standard deviation).

^b^ nd: not detected.

Values represent the means ± standard deviation of triplicates; means followed by different uppercase letters in the same column differ statistically (*p* < 0.05) by the Tukey’s test.

All variants of OP analyzed exhibited no flavonoids or low contents of these compounds, which is in accordance with the results reported by other researchers who studied Brazilian propolis [8]. OP1, OP7, and OP2 presented concentrations of 0.33, 3.03, and 3.10 mg QE/g, respectively, whereas in the other variants of OP no flavonoids were detected (Table 2). Despite having the highest total phenolic content, OP1 showed the lowest quantified total flavonoid content.

### Scavenging of free radicals by OP

#### Synthetic free radicals

The results of free radical scavenging activity of all variants of OP assessed are shown in Table 2. OP4 exhibited the highest activity, of 1.24 and 0.38 μmol TE/mg for scavenging ABTS and DPPH synthetic radicals, respectively. OP6 presented the lowest activity, of 0.29 and 0.01 μmol TE/mg for ABTS and DPPH radicals, respectively. Even though these methodologies present similarities, since they use synthetic free radicals, i.e. not found in biological systems, a considerable antioxidant capacity was observed, and ABTS radical scavenging was always higher than DPPH in all variants of OP analyzed. A probable explanation for this phenomenon is the fact that ABTS is soluble in water as well as in organic solvents, allowing the antioxidant activity of hydrophilic and lipophilic compounds [30]. In contrast, DPPH free radical scavenging is usually assessed in organic environments, mainly alcoholic, which can restrain the antioxidant activity of some compounds [31,32]. It is worth mentioning that the EEP used in this study are soluble in 80% v/v ethanol. OP had a high positive correlation (0.95) both for DPPH and ABTS. This demonstrates a very similar action of the antioxidant compounds present in the seven variants of propolis tested and these free radicals. The same trend was observed in the correlation with TFC, which was 0.95 and 0.94 for ABTS and DPPH, respectively.

#### Reactive oxygen species (ROS)

ROS are free radicals in which an unpaired electron is found centered in the atom of oxygen. In biological systems, they are involved in energy production, phagocytosis, cell growth regulation, intercellular signaling, and synthesis of important biological substances. Nevertheless, excess ROS in cells can trigger harmful effects such as cancer, neurodegenerative diseases, anemia, ischemia, and low-density lipoprotein oxidation, the latter leading to cardiovascular problems [33].

All the seven variants of OP presented scavenging activity against ROO^●^ radical (Table 2). OP1 exhibited the highest activity, even though it was smaller than the standard gallic acid used as positive control (9.19 μmol TE/mg). Since the ratio between ORAC (ROO^●^) values and TPC (ORAC/TPC) is more adequate to evaluate antioxidant activity [34], it has been used to classify foods such as fruits, vegetables, and nuts in four groups (0–5, 5–10, 10–15, and > 15). ORAC/TPC ratios for all variants of OP assessed were higher than 15, indicating a strong and positive linear correlation between TPC and antioxidant activity.

All variants of OP presented high reactive species scavenging capacity against O_2_^●-^ radical, and OP4 and OP5 were the most active (Table 2). All variants of OP exhibited IC_50_ much smaller than the ones found for the standards epicatechin (227 μg/mL) and gallic acid (75.48 μg/mL), showing high capacity of deactivating superoxide radical. *Cynodon dactylon* extract, usually used in popular medicine, presented IC_50_ of 430.06 μg/mL [35], a value 140 times higher than that obtained for OP6, the variant that presented the lowest activity (IC_50_ = 2.91 μg/mL), and 1480 times higher than that found for OP5, the variant that presented the lowest activity against this radical (IC_50_ = 0.29 μg/mL).

IC_50_ values for scavenging HOCl were very similar in all variants of OP, except in OP6, which displayed the lowest activity and was statistically different from all the other variants (Table 2). However, all variants of OP analyzed, except OP6, showed low IC_50_ values compared to that found for the standard epicatechin (0.81 μg/mL) and lower than that registered for gallic acid (8.46 μg/mL). Therefore, OP can be considered an excellent HOCl scavenger, since lipoic acid, a natural antioxidant and cofactor for mitochondrial enzymes, presented IC_50_ of 1.2 μg/mL [36].

#### Correlation between antioxidant capacity, synthetic radicals, and ROS

Strong positive correlation was registered for ABTS x DPPH (0.95). Moderate correlations were found for DPPH x peroxyl radical (0.81), DPPH x hypochlorous acid (0.77), DPPH x superoxide radical (0.85), ABTS x peroxyl radical (0.75), ABTS x hypochlorous acid (0.72), and ABTS x superoxide radical (0.74). Therefore, the evaluation of the antioxidant capacity using synthetic radicals was not accurate enough to estimate the deactivation capacity of ROS, whose results demonstrate the *in vivo* biological potential better [37].

### Antimicrobial activity

MIC results obtained for all variants of OP against *S*. *mutans*, *S*. *oralis*, *S*. *sobrinus*, *S*. *aureus*, *P*. *aeruginosa*, and *E*. *coli* are shown in Table 3. OP1, OP2, and OP3 presented the smallest MIC against the Gram-positive bacteria *S*. *mutans*, *S*. *oralis*, *S*. *sobrinus*, and *S*. *aureus* (ranging from 12.5 to 100 μg/mL). OP2 exhibited the smallest MIC (25–50 μg/mL) to inhibit the growth of *S*. *sobrinus*.

**Table 3:** Minimal inhibitory concentration (MIC) and minimal bactericidal concentration (MBC) of ethanolic extracts of propolis (EEP), obtained from seven variants of Brazilian organic propolis (OP1–OP7), against *Streptococcus mutans*, *Streptococcus oralis*, *Streptococcus sobrinus*, *Staphylococcus aureus*, *Pseudomonas aeruginosa*, and *Escherichia coli*.

OP	*S*. *mutans*		*S*. *oralis*		*S*. *sobrinus*		*S*. *aureus*		*P*. *aeruginosa*		*E*. *coli*	
	MIC	MBC	MIC	MBC	MIC	MBC	MIC	MBC	MIC	MBC	MIC	MBC
	(μg/mL)											
**1**	50–100	400–800	12.5–25	100–200	50–100	>1,600	50–100	800–1,600	100–200	>1,600	>1,600	>1,600
**2**	50–100	>1,600	12.5–25	100–200	25–50	>1,600	50–100	>1,600	100–200	>1,600	>1,600	>1,600
**3**	50–100	800–1,600	12.5–25	200–400	50–100	>1,600	50–100	>1,600	100–200	>1,600	>1,600	>1,600
**4**	50–100	>1,600	25–50	400–800	50–100	>1,600	100–200	800–1,600	100–200	>1,600	>1,600	>1,600
**5**	100–200	>1,600	25–50	400–800	400–800	>1,600	100–200	>1,600	400–800	>1,600	>1,600	>1,600
**6**	400–800	>1,600	25–50	200–400	400–800	>1,600	400–800	>1,600	800–1,600	>1,600	>1,600	>1,600
**7**	100–200	400–800	25–50	100–200	100–200	>1,600	100–200	>1,600	200–400	>1,600	>1,600	>1,600

Ethanol 4% was used as negative control.

Brazilian propolis types 3 (from southern Brazil, produced from *Populus* sp.) and 12 (from southeastern Brazil, produced from *Baccharis dracunculifolia*) inhibited the growth of *S*. *mutans* at the concentrations of 25–50 μg/mL and 200–400 μg/mL, respectively [38]. MIC of red propolis against *S*. *aureus* ATCC 25923 was assessed and values ranging from 62.5 to 125 μg/mL were found [3]. Although the variants of OP herein tested have been collected in another region of the country and their chemical composition is completely different from that of Brazilian propolis types 3 and 12, they possess high antimicrobial potential, comparable to other Brazilian propolis, and therefore deserve attention in a future prospection of their active compounds.

OP1, OP2, OP3, and OP4 presented the best results of inhibition against *P*. *aeruginosa* (100–200 μg/mL). MIC values for *E*. *coli* were not found at the tested concentrations in the present work (> 1,600 μg/mL). MIC of Turkish propolis against *P*. *aeruginosa* was assessed and the value of 1,250 μg/mL was found, but MIC against *E*. *coli* was not detected up to the maximum concentration tested (> 10,000 μg/mL) [39]. The antibacterial activity of propolis is higher against Gram-positive bacteria due to the presence of flavonoids, acids, and aromatic esters in resin. These compounds have effects on the cell walls of these microorganisms through a mechanism still not known [40,41]. Given that Gram-negative bacteria have two plasma membranes [42], the outer one covering the cell wall, we can hypothesize that OP phenolic acids could not penetrate the outer membrane and, therefore, were not able to reach their target. Since Gram-positive bacteria do not have the outer membrane [42], OP could not act against their cell wall. Further studies should be conducted to test this hypothesis.

In order to have a complete antimicrobial activity profile, tests for determining MBC of all variants of OP assessed were performed (Table 3). MBC results for Gram-positive bacteria ranged from 100 to > 1,600 μg/mL. OP1 and OP7 presented the smallest MBC for *S*. *mutans* (400–800 μg/mL), OP1, OP2, and OP7 for *S*. *oralis* (100–200 μg/mL), and OP1 and OP4 for *S*. *aureus* (800–1,600 μg/mL).

The crude extract of any natural product that exhibits MIC lower than 500 μg/mL is promising and deserves further investigation to elucidate its mechanism of action [43]. Therefore, the low MIC values registered for all variants of OP tested in this study demonstrate that they are promising antimicrobial compounds.

Different types of Brazilian propolis are among the most studied propolis around the world. Several compounds with different biological properties, which have been correlated with antimicrobial activity, have already been isolated from them, such as *tt*-farnesol, apigenin, vestitol, and neovestitol [44,45]. Taking into consideration that the OP analyzed are poor in flavonoids, the phenolic acids present in the samples studied are probably related to antimicrobial/antioxidant activity [46]. Antibiotics have recently been proven to cause oxidative stress on bacteria, thus affecting their survival [47,48]. Therefore, since the OP variants assessed in this study display antioxidant properties, their antimicrobial effects are probably not due to oxidative stress. Although Brazilian propolis has been proven to display antimicrobial activity, this might be related to the inhibition of proton pump [49] or to virulence factors [50], neither of which is related to antioxidant activity. However, further experiments should be performed to elucidate OP antimicrobial mechanisms of action.

MIC values found for green and red Brazilian propolis against *S*. *mutans* were 400 and 100 μg/mL, respectively [51,38,44]. Based on these MIC values, all variants of OP herein tested may have remarkable anticaries effects. Further studies should be conducted to isolate the compound(s) responsible for their antimicrobial property, mainly because this is the first report on organic propolis. Also, the low MIC value against *P*. *aeruginosa* exhibited by all variants of OP analyzed is outstanding because, to the best of our knowledge, this is the first type of Brazilian propolis to show ability for inhibiting the growth of this microorganism.

**Fig 2:**
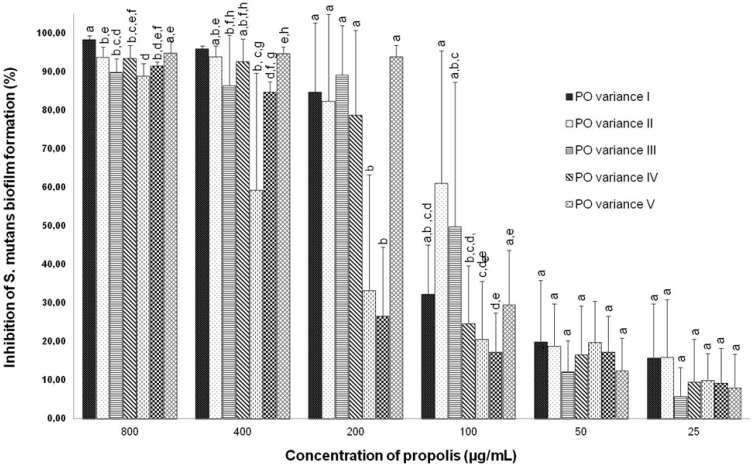
Inhibition of *Streptococcus mutans* biofilm formation by seven variants of organic propolis (OP1–OP7). Statistical analysis performed using ANOVA followed by Kruskal-Wallis test. Means followed by different letters differ statistically at the same concentrations of different variants of OP (*p* ≤ 0.05).

Since all variants of OP tested showed low MIC values against *S*. *mutans* (ranging from 50 to 800 μg/mL, Table 1), we decided to investigate their potential ability to inhibit *S*. *mutans* biofilm formation (Fig 2). According to the results, all variants of OP were able to inhibit *S*. *mutans* biofilm formation at the concentrations of 400 and 800 μg/mL (about 90%, except OP5 at 400 μg/mL). However, at the concentration of 200 μg/mL, OP5 and OP6 did not show good inhibition. The best performance to inhibit *S*. *mutans* biofilm formation (about 60% and 50%, respectively) at the concentration of 100 μg/mL was exhibited by OP2 and OP3.

Taking into account that the essential oils are well-recognized for their antimicrobial and antibiofilm properties [23,43,52], we compared the results of essential oil fractions to the crude extracts of OP and observed that OP2 and OP3 are promising antibiofilm agents. This comparison is very interesting, because the crude extracts of OP can be fractioned, which may intensify their antibiofilm properties by increasing the concentration of the active compounds.

### Anti-inflammatory activity

**Fig 3:**
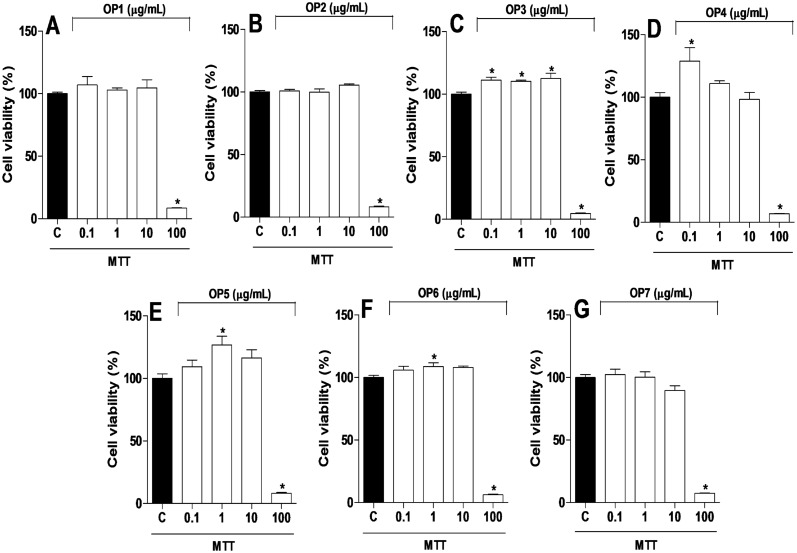
Citotoxicity effect of seven different organic propolis on macrophages. The groups were: control (vehicle) and organic propolis (OP1–OP7) at the concentrations 0.1, 1, 10, and 100 μg/mL. Data were expressed as means ± SD using ANOVA followed by the Tukey’s test. The results were considered statistically significant when *p* < 0.05. *p < 0.05 compared to the control group (C).

The MTT viability test was used to determine the cytotoxicity effect of seven different EEP in a RAW 264.7 macrophage cell line. The results showed that none of the EEP, at the concentrations of 0.1, 1, and 10 μg/mL, changed cell viability (*p* > 0.05) (Fig 3A–3G). Since the number of cells significantly decreased (*p* < 0.05) at the concentration of 100 μg/mL in all samples, the concentrations of EEP selected for the anti-inflammatory assays were 0.1, 1, and 10 μg/mL.

**Fig 4:**
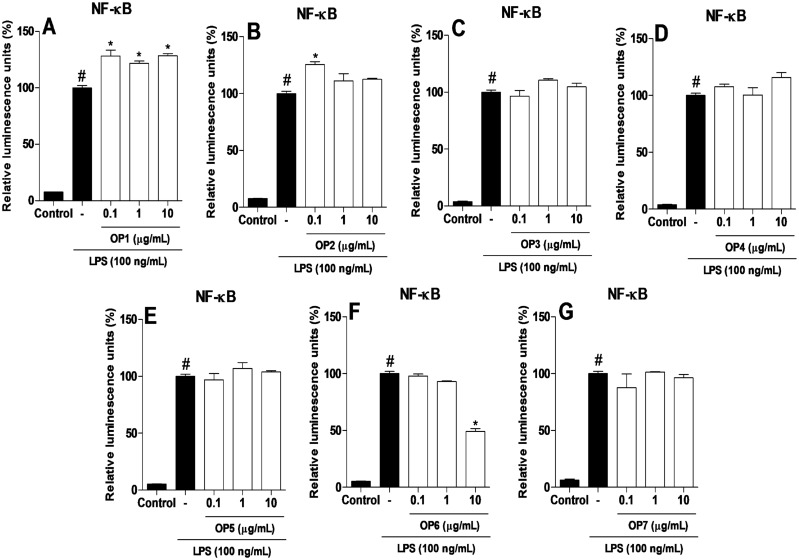
Activity of seven variants of organic propolis in the activation of NF-κB on macrophages. The groups were: control (vehicle) and organic propolis (OP1–OP7) at the concentrations of 0.1, 1, and 10 μg/mL. Data were expressed as mean ± SD using ANOVA followed by the Tukey’s test. The results were considered statistically significant when *p* < 0.05. #*p < 0*.*05 compared to the control group* (C); *p < 0.*05 compared* to LPS (–).

**Fig 5:**
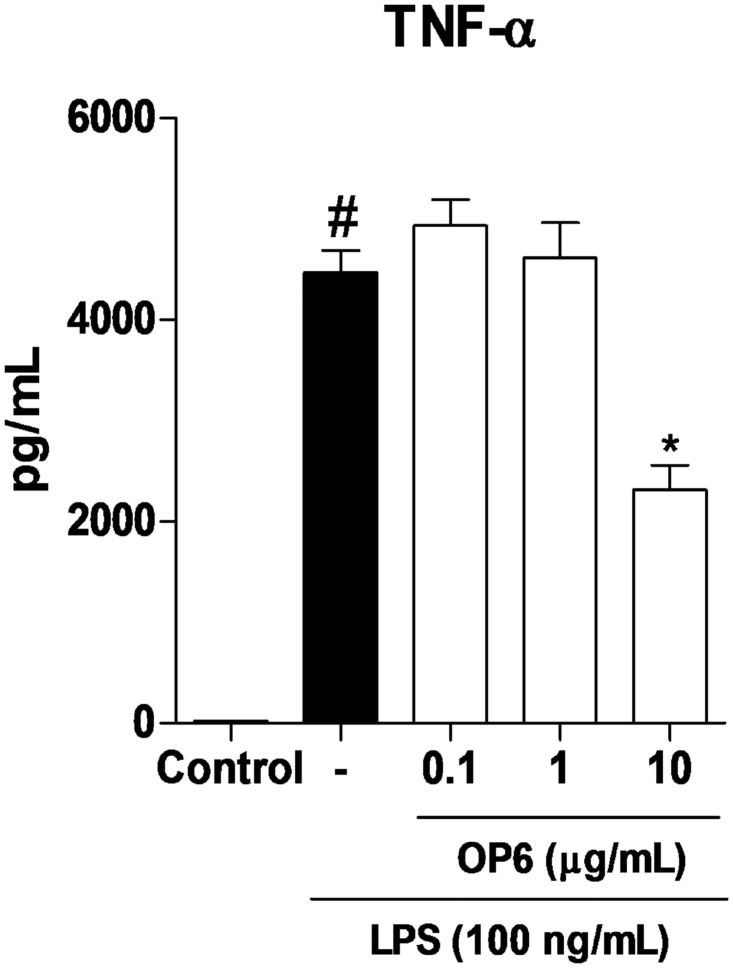
Activity of the ethanolic extract of organic propolis type 6 (OP6) in the release of TNF-α on macrophages. The groups were: control (vehicle) and OP6 at the concentrations of 0.1, 1, and 10 μg/mL. Data were expressed as mean ± SD using ANOVA followed by the Tukey’s test. The results were considered statistically significant when *p* < 0.05. #*p* < 0.05 compared to the control group (C); *p < 0.05 compared to LPS (–).

OP6 decreased NF-kB activation (Fig 4F) and TNF-α release (Fig 5) on macrophages at the concentration of 10 μg/mL compared to the control group LPS (*p* < 0.05). However, OP1, OP2, OP3, OP4, OP5, and OP7 did not modulate NF-kB activation compared to LPS (*p* > 0.05) (Fig 4B, 4C, 4D, 4E and 4G). Brazilian green propolis (state of Minas Gerais, southeastern Brazil) strongly decreased NF-κB activation at the concentrations of 100 and 300 μg/mL [53], which is similar to our findings for OP6 at an even lower concentration (10 μg/mL). Also, a type of propolis from Botucatu (state of São Paulo, southeastern Brazil) displayed anti-inflammatory activity [54]. The main finding of this study was the fact that OP6 decreased proteins MAPK p-p38 and p-JNK phosphorylation and NF-κB activation in RAW 264.7 macrophage cells at the concentration of 10 μg/mL, results that are in accordance with the studies cited. Based on our findings, OP6 is a promising natural product that inhibits the NF-κB pathway at low concentrations (10 μg/mL). Additional studies should be carried out to determine the genes and proteins that affect this important pathway of the inflammatory process. Furthermore, inasmuch as OP6 has a great potential for the discovery of novel bioactive molecules with anti-inflammatory activities, further studies are needed for the isolation and identification of bioactive compounds present in this type of propolis.

## Conclusions

This study allowed the identification of the antioxidant, antibacterial, and anti-inflammatory potential of certified Brazilian organic propolis. OP1, OP4, and OP5 stood out for displaying antioxidant activity. Also, OP in general exhibited strong antimicrobial activity against Gram-positive bacteria such as *S*. *mutans*, *S*. *oralis*, *S*. *sobrinus*, and *S*. *aureus*, as well as good inhibition of both *S*. *mutans* biofilm formation and Gram-negative bacteria growth, mainly *P*. *aeruginosa*. All variants of OP assessed greatly inhibited biofilm formation, and OP6 showed the highest anti-inflammatory activity. Gallic acid was found in all variants of OP studied, whereas artepillin C and pinocembrin were detected only in OP4 and OP7, respectively. Therefore, additional studies on fractionation and isolation of the bioactive compounds of these OP are necessary, which may lead to the discovery of compounds with potential use in pharmaceutical industries, as well as in the development of functional food products.
